# Phytochemical Fingerprinting and In Vitro Bioassays of the Ethnomedicinal Fern *Tectaria coadunata* (J. Smith) C. Christensen from Central Nepal

**DOI:** 10.3390/molecules24244457

**Published:** 2019-12-05

**Authors:** Shyam Sharan Shrestha, Stefania Sut, Serena Barbon Di Marco, Gokhan Zengin, Valentina Gandin, Michele De Franco, Deepak Raj Pant, Mohamad Fawzi Mahomoodally, Stefano Dall’Acqua, Sangeeta Rajbhandary

**Affiliations:** 1 Department of Botany, Tribhuvan University, Kirtipur 44618, Nepal; shyamsharan999@gmail.com (S.S.S.); drpant.agbot@gmail.com (D.R.P.); s.rajbhandary@cdbtu.edu.np (S.R.); 2 DAFNAE, Department of Agronomy, Food, Natural Resources, Animals and Environment, Agripolis Campus, University of Padova, 35020 Legnaro (PD), Italy; stefania_sut@hotmail.it; 3 Department of Pharmaceutical and Pharmacological Sciences, University of Padova, Via Marzolo 5, 35131 Padova, Italy; serena.barbondimarco@studenti.unipd.it (S.B.D.M.); valentina.gandin@unipd.it (V.G.); miky_defra@hotmail.com (M.D.F.); 4 Department of Biology, Science Faculty, Selcuk University, Campus, Konya 42130, Turkey; gokhanzengin@selcuk.edu.tr; 5 Department of Health Sciences, Faculty of Science, University of Mauritius, 230 Réduit, Mauritius; f.mahomoodally@uom.ac.mu

**Keywords:** *Tectaria coadunata*, antioxidant, medicinal fern, chromatography, procyanidins, flavonols

## Abstract

*Tectaria coadunata*, an ethnomedicinal fern used in Nepal to treat a large number of diseases, has been poorly studied with regard to its phytochemical composition and possible bioactivity. This study was performed with the aim of supporting traditional medicine as a new source of bioactive constituents. Phytochemical compositions of methanol extracts were determined by nuclear magnetic resonance (NMR), liquid chromatography–diode array detector–mass spectrophotometry (LC-DAD-MS), and liquid chromatography–fluorescence–mass spectrometry. Quali-quantitative data revealed large amount of procyanidins, mainly of the A-type, as well as eriodictyol-7-*O*-glucuronide and luteolin-7-*O*-glucoronide as main constituents. The antioxidant, cytotoxic, and inhibitory activity of five enzymes that are implicated in human diseases was evaluated for the extract and fractions. High free-radical scavenging activity in 2,2-diphenyl-1-picrylhydrazyl (DPPH) and 2,2′-azino-bis(3-ethylbenzothiazoline-6-sulphonic acid) (ABTS) assays and inhibitory activities against cholinesterases and tyrosinase were observed. Furthermore, a moderate cytotoxic effect was observed on the 2008 and BxPC3 cell lines. Overall results showed potential usefulness of this fern as a source of phytochemicals for pharmaceutical uses.

## 1. Introduction

The use of medicinal plants has been rooted in Nepalese culture since ancient times, but this traditional knowledge is threatened by the loss of oral tradition and the use of synthetic drugs. The richness of Nepalese flora is well described and is mainly related to the peculiar geographic and pedoclimatic conditions of this country, characterized by large altitudinal variations ranging from nearly 59 m up to 8848 m in height (Mount Everest). In this context, the exploration of Nepalese flora used in traditional medicine is a unique opportunity to study bioactive extracts as sources of new natural products. The ethnopharmacology of several species is still poorly considered and can offer new research opportunities. In rural areas of Nepal people are still dependent on medicinal plants because of the scarce availability and high costs of synthetic medicines [1]. In fact there are 8.4 million indigenous people living in different parts of Nepal that rely on traditional medicine. The study of ethnopharmacology is fundamental to providing scientific support to the users, to preserve the knowledge in this field, and also to offer the opportunity to explore scarcely studied medicinal plants. It has been calculated that 1792–2331 medicinal and aromatic plants are used for different diseases. In Nepal, 293 species of fern and its allies have been reported [2]*,* one of which is *Tectaria coadunata*, a terrestrial fern 30–100 cm tall. Its rhizome is creeping and erect, thick, and densely scaled, and its color is dark brown. Fronds clustered and stipe are stramineous to pale, glossy, and glabrescent. A sorus is found on each side of mid rib in a single row at the vein. Indusia are large, brown, entire, clypeate, membranous, glabrous, or hairy [3]. It is found in dense forests ranging from 500 to 2500 m in Chinese regions including Guangdong, Guangxi, Guizhou, Sichuan, Taiwan, Xizang, and Yunnan. It is also found in other countries such as Bhutan, India, Laos, Malaysia, Myanmar, Nepal, Sri Lanka, Thailand, Vietnam, and Madagascar. The plant is widely distributed in north-facing slopes.

*T. coadunata* is used to treat various ailments such as stomach pain and giardiasis [4], gastrointestinal disorders, diarrhea and jaundice [5], and to eradicate worms. Leaves are crushed and juice applied to the cut wounds to stop bleeding. The rhizome of *T. coadunata* is used for its anthelmintic activity and against stomach pains and gastrointestinal disorders. During diarrhea and dysentery, leaves are taken and prepared as vegetables, or about three spoons of root paste are taken in half a glass of water twice a day after filtration with cotton cloth for 3–4 days. Today this species is not cultivated in Nepal but is largely used in herbal medicinal practice.

Previous chemical investigations on *T. coadunata* are limited to a single paper in which 21 compounds were detected by gas chromatography–mass spectrophotometry (GC-MS) analysis [6]. The constituents have been indicated to involve general classes of compounds such as carbohydrates, tannins, phenols, anthraquinone glycosides, coumarin glycosides, flavonoids, and steroids [7].

The present paper aimed to study *T. coadunate*, bridging the gap between its common medicinal use in Nepalese traditional medicine and the scarce knowledge related to its constituents and possible bioactivity. In this paper, comprehensive analytical approaches have been applied. Nuclear magnetic resonance (NMR) on crude extract, as well as liquid chromatography–diode array detector–mass spectrophotometry (LC-DAD-FLD-ESI-MS) and chromatographic separations were performed to collect information on phytochemical composition and to isolate the most abundant constituents. In vitro antioxidant and enzymatic assays on five enzymes that are implicated in human diseases were evaluated for the extract and fractions. Preliminary cytotoxicity assays on tumor cell lines were performed to assess potential activities of the extract, offering new information on this medicinal plant. Overall results aimed to assess the potential importance of such a plant as source of bioactive constituents and to explore its chemical constituents, offering a starting point for further studies focused on supporting the traditional uses with scientific data. Moreover, chemical characterization offers the opportunity to use the identified constituents as a marker compound to prepare standardized herbal remedies such as herbal teas with antioxidant properties or tinctures or dry extracts to be used as more concentrated supplements.

## 2. Results and Discussion

### 2.1. NMR Analysis of *Tectaria coadunata* Extracts

As starting point for extract characterization, NMR was chosen as a technique due to its ability to detect all kinds of phytoconstituents and their response factors, which are independent depending on the detected compound, following procedures previously used for other plant extracts [8,9]. Due to the complex composition of the extracts, some partially fractionated extracts have also been considered in order to simplify samples.

**Figure 1:**
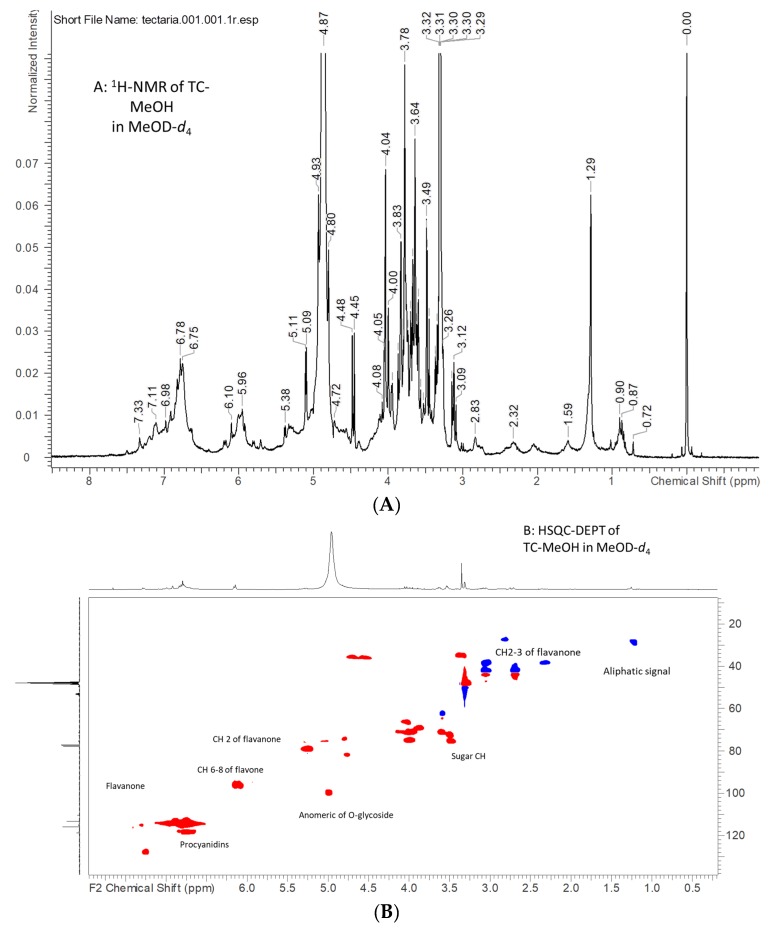
^1^H-NMR of *Tectaria coadunata* (TC)-MeOH (**A**) and heteronuclear single quantum coherence spectroscopy–distortionless enhancement by polarization transfer (HSQC-DEPT) of TC-MeOH (**B**) in MeOD-*d*_4_.

Concerning *T. coadunata* (TC)-MeOH, this extract may represent in large part the whole phytocomplex of the plant due to the efficacy of methanol in dissolving and extracting secondary metabolites from plant materials. The ^1^H-NMR spectrum presents two broad signals in the deshielded proton region, one being δ 6.50–7.50, ascribable to aromatic signals of phenolics, and another in the range δ 5.85–6.20, supporting other less deshielded aromatics or double bonds. Further signals are the two doublets at δ 5.11 (*J* = 3.95) and 4.48 (*J* = 7.75) and several multiplets in the spectral region from δ 3.20 to 4.10; all these signals suggest the presence of carbohydrates (Figure 1). The aliphatic region of the spectrum appears less populated, presenting a group of multiplets that can suggest the presence of aliphatic chains. Protons of the sugar part can be ascribed to saccharose and glucose on the basis of comparison with the spectral database.

To increase the signal intensity of secondary metabolites, partition with organic solvent was used and the TC-EtOAc extract was also analyzed. The ^1^H-NMR spectrum of this extract was more resolved compared to the TC-MeOH extract, allowing the observation of more sharp peaks in the aromatic and in the sugar regions as well as in the aliphatic parts. More information can be obtained combining the data obtained from the heteronuclear single quantum coherence spectroscopy–distortionless enhancement by polarization transfer (HSQC-DEPT) spectrum, allowing us to assign the value of the chemical shift of both H and C of all non-quaternary positions, and heteronuclear multiple quantum coherence (HMBC), which allows us to observe long-range correlations from H to C with a distance of 3–4 bonds. Further connectivity data were obtained from the correlation spectroscopy (COSY) spectrum, showing proton couplings. Data are summarized in Table 1 and Table 2, and allow us to substantiate the presence of different classes of constituents. Exemplificative spectra are reported in the Supplementary Materials. Thus, TC-MeOH mostly contains phenolics ascribable to flavone or catechin-type polymers such as procyanidins, and glycosides of flavonoids.

Fractionation with Sephadex allowed us to obtain a methanol-eluted fraction that appears to be mostly composed of eriodictyol-7-*O*-glucuronide. On the other hand, the acetone-eluted fraction resulted to be mostly formed of procyanidin fraction [10]. The diagnostic signals that support the presence of procyanidin are aromatic H-6 and H-8 for the upper units and H-6 for the lower units; furthermore, signals ascribable to position 2 of monomers were observed at δ_H_ 5.31 and δ_C_ 79.3 as well as δ_H_ 4.81 and δ_C_ 74.3, ascribable to position 2 of the lower units. Other relevant signals can be ascribed to other C-3 positions (Table 1) and signals ascribable to CH_2_ were detected at δ_H_ 3.14–2.72, δ_C_ 42.6, and δ_H_ 3.05, 2.39, and δ_C_ 37.4. A summary of main resonance ascribable to procyanidins in the TC-EtOAc fraction is shown in Table 2.

The overall NMR analysis on *T. coadunata* extracts revealed a composition mostly formed by glycosidic flavanone and procyanidins. Thus, further investigations were undertaken applying LC-MS-based approaches.

### 2.2. Quali-Quantitative Analysis

#### HPLC Coupled with Diode Array, Mass Spectrometry, and Fluorescence for the Analysis of Phenolic Constituents

**Figure 2:**
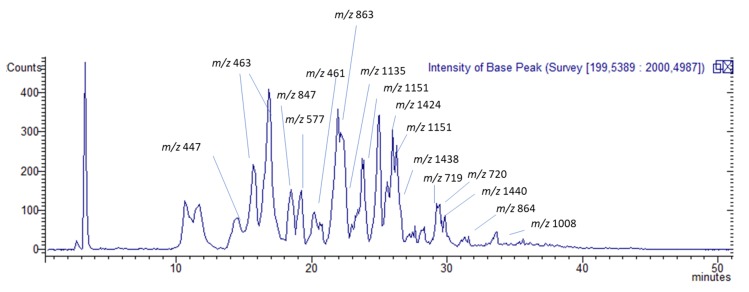
Base peak ion LC-MS chromatogram of TC-EtOAc showing the *m*/*z* values and corresponding peaks of identified compounds.

**Figure 3:**
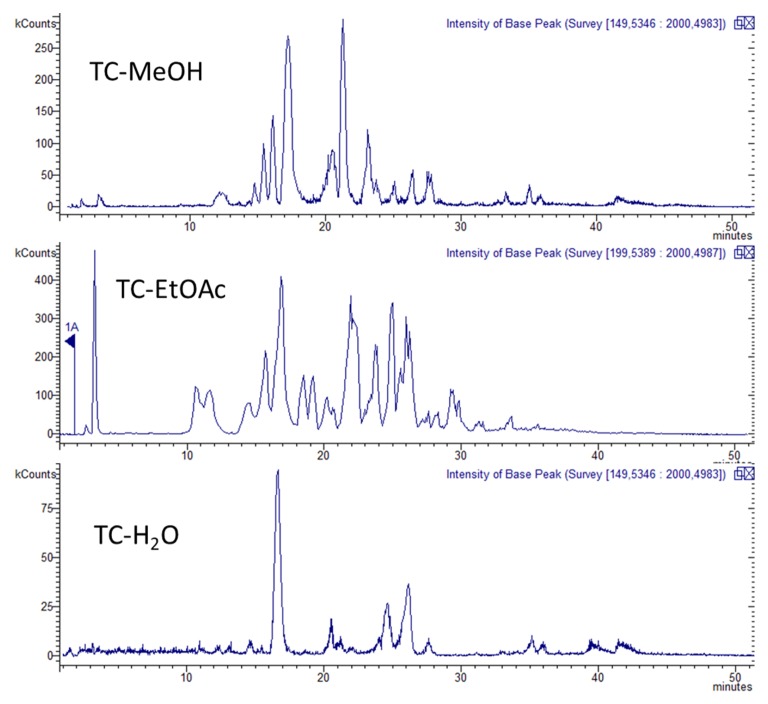
Base peak ion LC-MS chromatogram of TC-MeOH, TC-EtOAc, and TC-H_2_O extracts.

TC-MeOH, TC-EtOAc, and TC-H_2_O extracts of *T. coadunata* were analyzed by HPLC-DAD-MS, allowing the identification of secondary metabolites according to their retention time and mass fragmentation (Table 3, Figure 2). The three extracts, as shown in Figure 3, revealed the presence of procyanidins and glycosylated flavonoid and results were in agreement with NMR data. The identified flavonoids were namely naringenin-7-*O*-glucuronide, eriodictyol-7-*O*-glucuronide, and luteolin-7-*O*-glucuronide; for the latter two constituents, structures were elucidated by NMR and MS analysis after purification, while comparison with the reference standard confirmed the identity of the first compound. Procyanidin (PAC) dimers, trimers, and tetramers, as well as larger polymers, were detected in all the extracts. Identification of different PACs was tentatively done by MS data and revealed the presence of both A- and B-type PACs; in particular, the B-type procyanidin dimer was detected at 19.5 min and different A-type procyanidin trimers at 17.9, 19.1, and 20.8 min. From a quantitative point of view, when comparing the three extracts the dimers and trimers were more abundant in TC-EtOAc. An A-type procyandin tetramer and bigger polymers of both A and B types were mostly abundant in TC-MeOH. To assess the structures of procyanidin, purification by Sephadex was performed. The most abundant compound in TC-MeOH was an A-type procyanidin tetramer with one unit of (epi)afzelechin (25.2 mg/g), and the most abundant compound in the TC-EtOAc and the TC-H_2_O extracts was the A-type procyanidin trimer (38.69 mg/g and 2.58 mg/g, respectively).

Flavonoids are present in significant amounts in all three extracts, as reported in Table 4. TC-EtOAc contains higher amounts of flavonoid (24.28 mg/g). In TC-MeOH, flavonoids are present in an amount comparable to that of TC-H_2_O (2.70 and 1.74 mg/g), except for naringenin-7-*O*-glucuronide which in TC-MeOH is not detectable.

With regard to the quantitative analysis, PACs were analyzed using HPLC-HILIC-FLD and results are summarized in Table 4. An exemplifying chromatogram of methanolic extract is reported in Figure 2. TC-MeOH and TC-EtOAc fractions present the highest amount of total PACs (105.49 mg/g and 98.0 mg/g), accounting for more than 50% of total polymers in TC-MeOH and more than 50% of the total as trimers in TC-EtOAc. PACs are found in TC-H_2_O in low amounts (3.91 ± 0.5 mg/g), as expected due to their nature as poorly water-soluble compounds. PACs presenting lower molecular weights are more abundant in TC-EtOAc, while larger polymers concentrate in TC-MeOH. Since TC-EtOAc and TC-H_2_O extracts were obtained by the fractionation of TC-MeOH, we could argue that PACs with increasing molecular weight are less soluble in water and in ethyl acetate, and thus can precipitate during the liquid–liquid partition. Previous studies reported that, in methanol solvent, smaller PAC oligomers are more soluble than bigger ones because the solute surface area exposed to the extraction solvent is larger and thus more solute–solvent interactions are present [11].

Phytochemical composition of the TC-EtOAc revealed a composition rich in condensed tannins, with the presence of flavonols and dihydroflavone.

### 2.3. In Vitro Bioassays

#### 2.3.1. Antioxidant Activity

The interest in the pharmacological potential of medicinal plants might be due to their polyphenol compounds, in particular to flavonoids. From this perspective, some biological activities including the antioxidant activity of *T. coadunata* extracts were evaluated using total antioxidant capacity or phosphomolybdenum, radical scavenging (2,2-diphenyl-1-picrylhydrazyl (DPPH) and 2,2′-azino-bis(3-ethylbenzothiazoline-6-sulphonic acid) (ABTS)), reducing power (cupric-reducing antioxidant (CUPRAC) and ferric-reducing antioxidant power (FRAP)), and metal chelating assays.

Results of in vitro bioassays are reported in Table 5 with regard to antioxidant activity, and in Table 6 with regard to enzyme inhibition.

**Figure 4:**
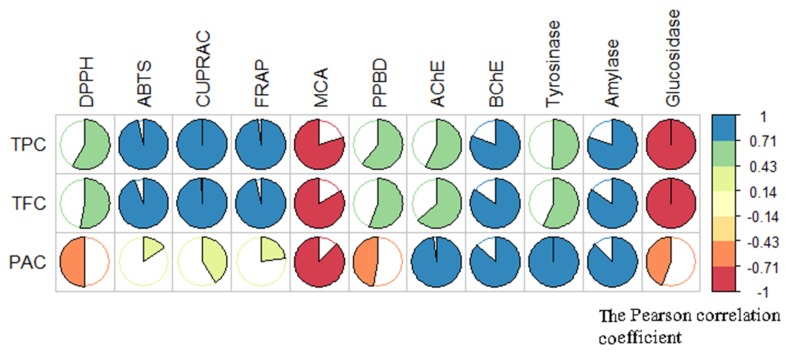
Correlation coefficients between total bioactive compounds and biological activities (Pearson correlation coefficient (R), *p* < 0.05). TPC: total phenolic content; TFC: total flavonoid content; PAC: procyanidin; PPBD: phosphomolybdenum assay. MCA: metal-chelating assay.

The free radical scavenging activity of *T. coadunata* was evaluated using the DPPH and ABTS radical scavenging assays. As shown in Table 5, the values of DPPH radical scavenging activity for the three extracts of *T. coadunata* range from 762.62 mg TE/g of TC-MeOH extract to 933.97 and 948.59 of TC-H_2_O and TC-EtOAc extracts, respectively. In ABTS radical scavenging activity *T. coadunata* ranges from 1097.10 mg Trolox equivalent (TE)/g of the TC-MeOH extract to 1661.21 of TC-EtOAc. Additionally, both in DPPH assay and ABTS, the TC-EtOAc extract had the highest radical scavenging activity among all samples, which is coherent with its high amount of polyphenols (276.70 mg GAE/g). This fact also was observed by correlation analysis and the analysis results are given in Figure 4. In any case, it is to be remembered that most TPC and DPPH assays can also present positive results with reducing sugars and other chemical constituents that can be subjected to oxidation other than phenolics.

The results obtained for *T. coadunata* are coherent with the literature; in fact, polyphenolics are well-known antioxidant agents. In particular, among secondary plant metabolites procyanidins are the most liable to oxidation, and their activity is closely related to plant defense systems against oxidative stress [12]. It has also been reported that the antioxidant activity depends on polymerization and increases with galloylation [13]. However, a previous paper [14] reported that the increase of the antioxidant activity is not directly proportional with the degree of polymerization, but relies mainly on the number of hydroxyl groups, which can increase as a consequence of polymerization. The major determinant for radical-scavenging capability is the presence of a catechol group in ring B that is able to reduce highly oxidizing free radicals such as superoxide, peroxyl, alkoxyl, and hydroxyl radicals by hydrogen atom donation [15].

In the light of this mechanism, it can be concluded that the high antioxidant activity of *T. coadunata* extract is due to its PAC content, and TC-EtOAc extract has the highest activity, presenting indeed the highest amount of PAC dimers (11.13 mg/g), trimers (57.79 mg/g), tetramers, and polymers (29.08 mg/g). Moreover, several investigations have shown that flavonoids such as epicatechin, catechin, and their related procyanidins can absorb through membranes through associations with the polar head groups of phospholipids, generating a flavonoid coat which would provide protection against oxidants as well as other external aggressors by limiting the access of oxidants to the bilayer and/or controlling the rate of propagation of free radical chain reactions occurring in the hydrophobic core membranes [16]. Particularly, galloylated catechins could affect the membrane configuration by forming more compact structures that limit the access of pro-oxidants [17].

Reducing power assays, namely FRAP (from Fe^3+^ to Fe^2+^), CUPRAC (from Cu^2+^ to Cu^+^), and phosphomolybdenum (from Mo (VI) to Mo (V)) assays, were performed to evaluate electron-donating abilities of the tested extracts and the results were similar to the results of radical scavenging assays (EtoAC > H_2_O > MeOH). The results can be attributed to the presence of phenolics, especially PAC. Our findings were also supported by several researchers who reported that phenolics have great potential as reducing agents [18,19]. In contrast to radical scavenging and reducing power assays, TC-EtOAc was not active in the metal-chelating assay. TC-H_2_O was more active than TC-MeOH. This contradictory finding could be explained with the non-phenolic chelators such as peptides or polysaccharides. This view is supported by Rice-Evans et al. [20] who reported that metal-chelating ability is a minor antioxidant property of phenolics.

#### 2.3.2. Test of Inhibitory Effect Against Degenerative and Metabolic Enzymatic Activities: Cholinesterases, α-Amylase, α-Glucosidase, and Tyrosinase

The inhibitory activities of tested extracts against cholinesterases (acetylcholinesterase (AChE) and BChE (butyrylcholinesterase)), α-amylase, α-glucosidase, and tyrosinase were tested since enzyme inhibition is considered as one possible strategy to manage some chronic conditions of health problems. A well-known treatment regime for Alzheimer’s disease includes cholinesterase inhibitors such as donepezil, galantamine, and rivastigmine. Current treatment modalities to manage type-2 diabetes consist of α-amylase and α-glucosidase inhibitors such as acarbose, miglitol, and voglibose. Epidermal tyrosinase inhibition by kojic acid is currently used to manage skin hyperpigmentation conditions. However, the adverse effects associated with the use of currently available enzyme inhibitors have fueled interest in finding novel therapeutic agents and this is why the extracts were tested in this context [8].

Generally, as presented in Table 6, TC-H_2_O demonstrated the lowest activity against all enzymes, while the TC-EtOAc extract presented the highest values, especially for AChE (6.22 mg GALAE/g), BChE (9.82 mg galantamine equivalent (GALAE)/g), and tyrosinase (153.89 mg GALAE/g) inhibition. TC-MeOH and TC-EtOAc showed prominent inhibitory effects against AChE (TC-EtOAc: 6.22 mg GALAE/g, TC-MeOH: 5.58 mg GALAE/g), which is consistent with the literature, indicating that phenolic compounds have cholinesterase inhibitory activities [21].

#### 2.3.3. Discussion of the Results of Acetyl and Butyril Cholinesterases Related to Phytochemical Composition of the *T. coadunata* Extracts

With respect to cholinesterase, the inhibitory activity was tested both on acetylcholinesterase (AChE) and butyrylcholinesterase (BChE), which are hydrolytic enzymes acting on acetylcholine (ACh) to terminate its actions in the synaptic cleft by cleaving the neurotransmitter to choline and acetate. Both enzymes are present in the brain and have been detected in neurofibrillary tangles and neuritic plaques. It was suggested that AChE predominates in the healthy brain, with BChE considered to play a minor role in regulating brain ACh levels [22]. Both enzymes represent legitimate therapeutic targets for ameliorating the cholinergic deficit considered to be responsible for the neurological decline characteristic of Alzheimer’s disease (AD). In this disorder, AChE activity decreases to only 33–45% of normal values as the disease progresses, while the activity of BChE increases by as much as 40–90% in certain brain areas, suggesting that this alteration of AChE to BChE ratio causes a change in the normally supportive role of BChE in hydrolyzing excess ACh. This implies that also BChE inhibition may serve as an appropriate therapeutic target to treat AD [23].

Results indicate significant AChE inhibitory activity for TC-EtOAc, and the non-detectability of nitrogen-containing compounds suggests the presence of non-alkaloidal inhibitors in the extract. The search for non-nitrogen containing AChE and BChE inhibitors is of interest since alkaloids lead to common side effects [24]. Furthermore, non-alkaloidal inhibitors probably have different types of interaction with the target enzyme due to the lack of a charged part, thus offering the opportunity to find other pharmacological properties [25].

In this regard a paper suggested that phenolic compounds are able to interact with amino acid residues defining the active site of AChE via a hydrogen bond, hydrophobic, and π-π interaction [26]. Multiple hydroxyl groups in the phenolic compound are believed to enhance the inhibitory action of AChE because of stronger binding capacity. These inhibitory actions explain the inhibitory potential of most of the phenolic compounds but not all follow the same mode of action. This fact was also confirmed by correlation analysis (Figure 4). Based on the correlation analysis, a strong correlation was found between total bioactive components and the inhibitory activities of cholinesterases (phenolics (R: 0.58 for AChE and R: 0.81 for BChE), flavonoids (R: 0.63 for AChE and R: 0.85 for BChE), and procyanidin (R: 0.98 for AChE and R:0.87 for BChE)).

The structural requirements of flavonoids as inhibitors of enzymes implicated in Azheimer’s disease, like AChE and BuChE, were previously investigated in combination with the established structure-activity relationships (SARs) of flavonoids as reactive oxygen species (ROS) scavengers and metal chelators. For example same flavonoids, such as quercetin, act as AChE and BuChE inhibitors, and docking experiments showed that they can efficiently bind with enzymes [27]. In particular, the presence of phenylchroman backbone present in flavonoids could be the reason behind AChE inhibition and, in addition, the position, number, substitution of hydroxyl groups, and the oxidation state of C-ring of the flavonoid structure could also determine the effectiveness of AChE inhibition. A close inspection revealed that the binding depends not only on the OHs at positions 5 and/or 7, but also on the catechol in ring B [26].

However, due to the complexity of polyphenolics and a limited understanding of their bioactivity, absorption, metabolism, and distribution to brain tissues, the development of effective polyphenolic compounds suitable for clinical application has been rather limited. In a previous study, Wang et al. demonstrated that fractions of procyanidins, namely monomers and oligomers, in vitro, interfere with the generation of soluble neurotoxic Aβ oligomer species implicated in neuronal dysfunction in AD [28]. However, in vivo studies on eight-week-old male Sprague Dawley rats placed on a polyphenol-free AIN-93M diet revealed that only the monomer (catechin derivative) was able to improve spatial memory function and to reduce Aβ-mediated neuropathology in the brain at a concentration of 400 nM following oral administration. This can be related to the bioavailability of monomeric (catechin type) derivatives and its metabolites, while oligomers are largely not bioavailable as intact molecules. Pharmacokinetic studies indicated that catechin and epicatechin glucuronides and methylated glucuronide metabolites are the most abundant metabolites after intake of polyphenol-rich fractions or extracts [28]. The same research group demonstrated that repeated dosing of monomers resulted in the accumulation of catechin and epicatechin metabolites in the brain with concentrations reaching >300 pmol/g. Moreover, a biosynthetic brain-targeted PAC metabolite, 3-*O*-methyl-epicatechin-5-*O*-β-glucuronide, at a physiologically relevant concentration, can significantly improve basal synaptic transmission and maintenance of long-term potentiation through mechanisms associated with activation of cAMP response element binding protein (CREB) signaling, a pathway involved in synaptic plasticity essential for learning and memory [28].

The potential central nervous system actions of flavonoids strongly depend also on their ability to enter the central nervous system (CNS) and so distribution studies in this tissue should be performed. It was observed that flavonoids of different sub-classes (flavanones and anthocyanins) are able to access and transverse the endothelial cell layer [29]. In addition, the major flavonoid metabolites found in the blood circulation, glucuronides and *O*-methylated derivatives, are also incorporated into endothelial cells, where they are deconjugated, forming aglycones which may then be able to enter glial cells and possibly the brain. For catechin and quercetin, the hypothesis of transport by diffusion can be raised due to their hydrophobicity, while for glucoside derivatives, this hypothesis is not reasonable due to the presence of the glucose moiety [30]. In this context, GLUT1 is a possible transporter, concurrent with the finding that intestinal GLUT2 may be involved in the transport of these compounds [30]. In another study by Faria et al., the isomers (+)-catechin and (−)-epicatechin were found to be capable of crossing the BBB layer, with, a significant difference between the transport of these two isomers reported, suggesting the involvement of a stereo-selective process [31]. This was demonstrated by studying the transport efficiency of 30 µM solution of catechin and epicatechin through rat brain endothelial cell (RBE4) in the presence of some transporter inhibitors. The results showed that phloridzin, an inhibitor of the sodium-dependent glucose transporter (SGLT1), affects only catechin transport, which is in agreement with a stereospecific effect. In the presence of cyclosporine A, a P-glycoprotein inhibitor, an increase in epicatechin transport, but not in catechin, was noted, suggesting the possibility that these compounds have different affinities for this transporter.

#### 2.3.4. Discussion of the Results of Amylase and Glucosidase Inhibitory Activity Related to Phytochemical Composition of the *T. coadunata* Extracts

Considering the other enzymatic activity reported in Table 6, amylase and glucosidase inhibition is quite low for each of the tested extracts, with values around 1 mmol acarbose equivalent (ACAE)/g for amylase inhibition and 5.5 mmol ACAE/g for glucosidase, demonstrating limited activity of *T. coadunata* extracts in this targets. This fact contrasts with the findings of several researchers who indicated significant inhibition abilities of PAC against amylase [32,33,34]. The contradictory results may be due to the antagonistic actions of phytochemicals in the extracts. At this point, the isolated compounds from *T. coadunata* could be individually tested as diabetic agents in further studies. 

All of the *T. coadunata* extracts (with the exception of the aqueous one) displayed tyrosinase inhibitory activity ranging from 149.41 mg kojic acid equivalent (KAE)/g for TC-MeOH extract to 153.89 mg KAE/g for the TC-EtOAc extract. This can be related to their high total phenolic content. Polyphenolics are able to act as cofactors or substrates of tyrosinase and in particular flavonoids containing a 3-hydroxy, 4-keto group, like eriodictyol, show significant tyrosinase inhibitory activity, which may be explained in terms of similarity with the di-hydroxyphenyl group in l-DOPA. Previously published data on flavonols indicated that aglycones but not their 3-glycoside derivates exhibit tyrosinase inhibitory activity, suggesting a role for the 3-hydroxyl group. However, this hydroxyl group may not be essential because several flavones, such as luteolin and luteolin 7-*O*-glucoside, which lack this 3-hydroxyl group, still present tyrosinase inhibitory activity [35].

### 2.4. Cytotoxicity Tests

Preliminary cytotoxicity tests were performed on two human tumor cell lines, 2008 (ovarian cancer) and BxPC3 (pancreatic cancer). Results are reported in Table 7.

TC-MeOH and TC-H_2_O exhibited a concentration of compound inhibiting cell growth by 50% (IC_50_) > 50 µg/mL on both cell lines, while TC-EtOAc showed a significant cytotoxicity, with a IC_50_ of 12.5 and 28.7 µg/mL against human pancreatic and ovarian cancer cell lines, respectively. This relevant activity of TC-EtOAc can be related to the content of PACs. Actually, the fractions with the highest degree of polymerization and galloylation have been reported in literature to exert the most toxic effect against cancer cells [13]. This result is in agreement with those of other authors who also attributed the greatest level of cytotoxicity to polyphenolic compounds with these characteristics [36]. Previous mechanistic studies suggested that procyanidins show different apoptotic mechanisms. In particular, procyanidins arrest BxPC-3 cells in the G1 phase, which is mediated by decreases of cyclin D1, E, A, and B1 and by an increase in the level of Cip1/p21, and inhibited MMP-2 expression. Thus, procyanidin treatment exerted anti-proliferative and anti-invasive effects in pancreatic cell lines, suggesting its application as a potent chemo-preventive or therapeutic agent for pancreatic cancer treatment [37]. On the other hand, previous studies in ovarian cancer cell lines showed that PACs exert cytotoxic activity via several mechanisms, inducing apoptosis with DNA damage and caspase-3 mediation; besides, down-regulation of pro-MMP-2 and a reduction in active MMP-2 levels imply a decreased invasive potential of the cells [38].

## 3. Materials and Methods

### 3.1. Plant Material

Plant twigs and rhizomes of *T. coadunata* were collected from Dakshinkali (27°36′24.88′′N and 85°15′40.01′′E), Kathmandu, Nepal. Elevation ranged from 1400 to 1509 m.

The plant twigs were dried carefully by proper pressing and the herbarium were prepared after mounting on the herbarium sheets. They were cross-checked with the herbarium deposited at the National Herbarium and Plant Laboratories, Godavari, Lalitpur (KATH). A voucher specimen was deposited with TU Herbarium number TC2018. Rhizomes were broken down into small pieces and air dried over 2–3 weeks inside the room until completely dried. The shade dried samples were grinded into fine powder with the help of an electric grinder. The powder obtained were preserved into zipper bag for extraction.

### 3.2. Extraction

The powder of plant material were subjected to extraction using methanol through percolation with intermittent sonication. Here, 50 g of plant powder were extracted three times with 500 mL of methanol such that the ratio of solvent in volume (mL) to the weight (g) of plant material would be 10:1. Then the solution was subjected to intermittent sonication for 2 h, i.e., continuous cycle of sonication at 30 kHz for 30 min (with 10 min interruption). After the completion of the cycle the solution was filtered with Whatman no.1 filter paper (Whatman Ltd., Kent, UK) and the filtrate was then subjected to evaporation at reduced pressure in rotary evaporator (IKA RV 10). The concentrated extract thus obtained was transferred to clean, dried and weighed glass vials. The obtained extract was called methanol extract (TC-MeOH). The resulting dried extract was then sealed and stored at 4 °C until use.

### 3.3. Isolation of Main Constituents

The TC-MeOH (15 g) was separated through a silica gel column chromatography, using 1% methanol in chloroform as mobile phase. Different fractions were obtained, analyzed by TLC using as eluents EtoAC:cyclohexane 2:1 and chloroform/methanol 99:1, and those presenting similar behavior were pooled. One-hundred fractions of 20 mL were collected and pooled on the basis of the chromatographic behavior. The fractions presenting similar TLC (fractions 23–45) spots were pooled. A liquid–liquid partition was performed with ethyl acetate and water, respectively, obtaining two different fractions after solvent removal: 3.15 g of ethyl acetate extract (TC-EtOAc) and 11.83 g of aqueous extract (TC-H_2_O). In order to assess the structure of the most abundant compounds, the aqueous extract (8 g) was separated by a Sephadex column eluting with methanol–water 50% (0.5 mL/min), column volume 2 cm × 40 cm. Fractions were pooled on the basis of their TLC behaviors. Further preparative HPLC using Varian 920-LC, equipped with column oven and UV-Vis detector was done. The separation was achieved through Agilent ZORBAX SB-C-18 (21.2 × 150 mm, particle size 5 μm) as stationary phase. The injection volume was 200 μL, the flow was 3 mL/min, and the temperature was set at 35 °C. The UV and Vis lamps were set at 280 and 454 nm, respectively. The mobile phase was 2% formic acid in water (A) and acetonitrile (B). A gradient program was used as follows: (0 → 30 min: A:B (95:5) → A:B (50:50) 30→ 50 min: A:B (50:50) → A:B (0:100) 50→ 55 min: A:B(0:100) → A:B (0:100) 55→60 min: A:B (0:100) → A:B (95:5)).

From water extract, luteoline-7-*O*-glucuronide was obtained (10 mg) and its structure was confirmed by 1D- 2D-NMR and using mass spectrometry. With the same protocol, from the ethyl acetate fraction (4 g) eridictiol-7-*O*-glucuronide (5 mg) was isolated, and its structure was confirmed by 1D 2D NMR and using mass spectrometry. NMR spectra were obtained on a Bruker Avance III 400 Ultrashield spectrometer with a superconducting 400-MHz magnet. Data were acquired in MeOD-*d*_4_ (Sigma-Aldrich) using Durian^®^ 4.95-mm NMR tubes (Durian Group). Chemical shifts are expressed in δ values in ppm. ^1^H-NMR and HSQC-DEPT, HMBC, and COSY experiments were acquired using standard Bruker sequences measuring p1 and d1 for each acquired sample.

### 3.4. Quali-Quantitative Analysis: HPLC HILIC-DAD-FLD-ESI-MS

Exact weights of methanolic, ethyl acetate and water (MeOH, EtOAc, and H_2_O) extracts (10 mg) were dissolved in 1 mL of methanol diluted 1:10 with the same solvent, and 1 mL was put in a vial; samples were prepared in triplicated.

In order to analyze MeOH, EtOAc, and H_2_O extracts, HPLC-DAD-FLD-ESI-MS was performed using a Chromatograph Agilent 1260 apparatus (Santa Clara, CA, USA) equipped with a 1260 autosampler, column oven, diode array detector (DAD), and fluorescence detector (FLD). After the column, the flow was separated by two “T” connectors: 50% of the liquid was split to DAD, 25% to FLD, and the other 25% to a Varian MS-500 ion trap mass spectrometer. Separation was achieved using a TOSOH TSK gel amide-80 (2.1 × 150 mm, particle size 3.5 µm) as stationary phase. The sample injection volume was 10 μL, the flow was 0.25 mL/min, and the temperature of column was set at 35 °C. UV–vis spectra were acquired in the range of 190–640 nm. The mobile phase was 1% formic acid in water (A) and acetonitrile (B). A gradient program was used as follows: (0 → 20 min: A:B (1:99) → A:B (20:80) 20 → 25 min: A:B (20:80) → A:B (20:80) 25 → 45 min: A:B (65:35) → A:B (65:35) 45 → 67 min: A:B (85:15) → A:B (1:99) 69 → 75 min: A:B (1:99) → A:B (1:99)). MS spectra were collected in the *m/z* 100–2000 range, using ESI ion source operating in negative ion mode. Fragmentation of the ionic species was obtained using the turbo data dependent scanning (TDDS) instrument function. Identification of compounds was obtained based on fragmentation spectra as well as the comparison with the literature and reference compounds, when available.

DAD and FLD detectors were used to estimate the amount of PACs and flavonoids and to acquire spectral data of eluted compounds. As reference compounds, PAC A2 (Sigma Aldrich, St. Louis, MO, USA) and luteolin (Sigma Aldrich) were used. The chromatograms were monitored in FLD for PACs (excitation 230 nm, emission 321 nm; scan range 200–500 nm), whereas flavonoids were monitored at 350 nm; UV–vis spectra were acquired in the range of 190–640 nm. Compounds quantification was obtained with the method of calibration curve: PAC A2 was used as external standard for PACs quantification, while luteolin was used for flavonoids. Calibration curves were as follows: y = 6.6721x + 8.6153 (R^2^ = 0.9991) for PAC A2; y = 127.77x − 2.4 (R^2^ = 0.9998) for luteolin.

### 3.5. Total Phenolic Content, Antioxidant, and Enzyme Inhibitory Assays

Total phenolic and flavonoid content (TFC) was detected by Folin–Ciocalteu colorimetric method [39]. Briefly, sample solution (50 µL) was mixed with the reagent of Folin–Ciocalteu reagent (100 µL, 1:9, *v*/*v*). The mixture was kept for 3 min at the room temperature and then sodium carbonate (75 µL, 2%) was added. The mixture was incubated for 2 h at the room temperature. After that, the absorbances were recorded at 765 nm. The results were expressed as standard compounds (gallic acid (GAE)).

For antioxidant capacity, chemical different assays including free radical scavenging (DPPH and ABTS), reducing power (CUPRAC and FRAP), ferrous ion-chelating (ferrozine method), and phosphomolybdenum assay were performed. The results were recorded on the basis of the spectrophotometric measurements. The methods details were described in our earlier paper [39]. To explain the results, we used standard equivalent way and thus Trolox and EDTA (for ferrous ion chelation) were selected as standards

For enzyme-inhibitory assays, we selected on some enzymes related global health problems, namely cholinesterases, tyrosinase, amylase, and glucosidase. The experimental procedures were given in our earlier paper [39]. Standard enzyme inhibitor compounds were used to evaluate the results. These compounds were galantamine (GALAE, for cholinesterases), kojic acid (KAE, for tyrosinase), and acarbose (ACAE, for amylase and glucosidase). All experimental procedures were performed with 96 wells microplate.

### 3.6. Cytotoxicity Studies

Ovarian (2008) and pancreatic (BxPC3) carcinoma cell lines were obtained from American Type Culture Collection (ATCC, Rockville, MD). Cell lines were maintained in the logarithmic phase at 37 °C in a 5% carbon dioxide atmosphere using RPMI-1640 medium (Euroclone) containing 10% fetal calf serum (Euroclone, Milan, Italy), antibiotics (100 units/mL penicillin and 100 g/mL streptomycin), and 2 mM l-glutamine.

The 3-(4,5-dimethyl-2-thiazolyl)-2,5-diphenyl-2*H*-tetrazoliumbromide) assay (MTT) was used as a relative measure of cell viability. Briefly, 10^3^ cells/well, dependent upon the growth characteristics of the cell line, were seeded in 96-well microplates in growth medium. After 24 h, the medium was removed and replaced with fresh medium containing the compound to be studied at the appropriate concentration (0.1–30 μM for isolated compounds, 1–100 μg/mL for EO). Triplicate cultures were established for each treatment. After 72 h, each well was treated with 10 μL of a 5 mg/mL MTT solution in phosphate-buffered saline (PBS) and, after 4 h of incubation, 100 μL of a sodium dodecylsulfate (SDS) solution in 0.01 M HCl were added. After an overnight incubation, the extent of MTT reduction was quantified spectrophotometrically using a microplate reader BioRad 680, by absorbance measurement at 540 nm. The mean absorbance for each drug dose was expressed as a percentage of the control, untreated, well absorbance and plotted vs. drug concentration. Cytotoxicity is expressed as the concentration of compound inhibiting cell growth by 50% (IC_50_). The IC_50_ values, the drug concentrations that decrease the mean absorbance at 570 nm to 50% of that of untreated control wells, were calculated using GraphPad Prism 4 (GraphPad Software, S. Diego, CA). The final value is the mean ± S.D. of at least three independent experiments performed in triplicate.

### 3.7. Statistical Analysis

The data of total phenolic content, antioxidant, and enzyme inhibitory assays were presented as mean ± SD and the statistical procedures were performed using GraphPad Prism 8 software. One-way ANOVA followed by Tukey’s multiple range was conducted to measure differences (*p* < 0.05) between the tested samples. The correlation values (Pearson’s correlation coefficients) between total components (TPC, TFC, and PAC) and biological abilities (antioxidant and enzyme inhibitory properties) were determined using R software v. 3.6.1.

## 4. Conclusions

In this paper the composition of the ethnomedicinal fern *T. coadunata,* spontaneously grown and collected in Nepal, was considered and the phytochemical investigations revealed significant amounts of procyanidins with different degrees of polymerization (with the exceptions of eriodictyol-7-*O*-glucuronide, luteolin-7-*O*-glucuronide, and naringenin-7-*O*-glucuronide). To assess the potential usefulness of the extract, in vitro antioxidant tests as well as inhibitory tests on five enzymes that are related to degenerative and metabolic diseases were explored, showing significant activities. In particular, AChE, BChE, tyrosinase, and amylase inhibitory activities were observed, and TC-MeOH and TC-EtoAc resulted more active compared to TC-H_2_O. These observations suggest that the higher amount of polyphenols in the two first extracts could be partly related to the measured activities. Results indicated that *T. coadunata* extracts, being a significant source of phenolics, could be considered for further studies aimed at developing co-treatments for Alzheimer’s disease using more complex in vitro and in vivo tests due to their significant antioxidant activity and partial target enzyme inhibition. In addition, their significant antioxidant effect could be an advantage in the management of metabolic diseases. Assays studying the anti-inflammatory effect of the extracts and isolated compounds may be added and implemented. Furthermore, significant cytotoxic activity was observed for ethyl acetate extract, mainly with respect to pancreatic cancer cells. Some of the constituents could thus be further studied, and research on the purified constituents will aid in assessing their potential usefulness in the therapeutic field. This medicinal fern could be suitable for cultivation in Nepal with the aim of obtaining plant material for the extraction of bioactive fractions for medicinal and pharmaceutical purposes.

## Figures and Tables

**Table 1:** Nuclear magnetic resonance (NMR) assignments of TC-MeOH. Data are obtained from H, heteronuclear single quantum coherence spectroscopy-distortionless enhancement by polarization transfer (HSQC-DEPT), correlation spectroscopy (COSY), and heteronuclear multiple quantum coherence (HMBC) spectra in MeOD-*d*_4_.

			TC-MeOH
δ_H_	δ_C_	Correlations	Assignments
			
7.45	115.3	150.5, 125.3	Aromatic phenol ring of procyanidin or tannin
7.31	114.5		Aromatic phenol ring of procyanidin or tannin
6.76	114.5	129.4, 118.7, 116.5	Aromatic phenol ring of procyanidin or tannin
			
7.25	127.7	156.8, 125.7, 79.5	Flavanol moiety
6.90–6.99	114.2–117.2	144.7, 118.5, 79.5	Flavanol moiety
6.16–6.15	94.5–95.8	196.3, 163.5, 103.5, 94.2	Flavanol moiety position H-6/8
5.26	78.3	196.3, 128.5, 113.5	Flavanol moiety CH position 2
2.47 dd		196.3, 127.8, 79.5	Flavanol moiety CH_2_ position 3
3.11 dd		196.3, 127.8, 79.5	Flavanol moiety CH_2_ position 3
			
5.01	98.9	163.4	Anomeric proton of O-glycoside residue
4.77	80.5		Flavonol or procyanidin CH
3.50	74.1–72.2	98.6, 75.6	Sugar residue CH
3.62	71.3		Sugar residue CH
3.86	68.8		Sugar residue CH
4.00	70.0		Sugar residue CH
4.05	74.6	70.0	Sugar residue CH
4.02	66.7	75.0	Sugar residue CH
2.32	37.3	172.6	Organic acid CH_2_
1.25	28.6		Aliphatic

**Table 2:** NMR assignments of TC-EtOAc. Data were obtained from H, HSQC-DEPT, COSY, and HMBC spectra in MeOD-*d*_4._

			TC-EtOAc
δ_H_	δ_C_	Correlations	Assignments
7.28	126.5	144.5, 119.4	aromatic phenol ring of procyanidin or tannin
7.00	115.0	144.5, 120.0, 73.6	catechin moiety H-2′ or H-6′
6.80	118.2	144.0, 129.4, 116.5	catechin H-5′
5.98–6.01	95.0–93.0	156.0, 101.0, 93	H-6/8 of catechin units
5.31	79.3		H-2 of upper unit of catechin/epicatechin moieties
4.81	74.2	67.5, 113.3, 119.8, 129.5	H-2 of lower units of catechin/epicatechin moieties
4.07	74.7		H-2 of lower units of catechin/epicatechin moieties
3.84	68.9	101.5, 37.4, 38.5	H-3 of upper units of catechin/epicatechin moieties
3.31	47.6		C-4 of upper units of catechin/epicatechin moieties
3.14–2.72	42.6		C-4 of terminal units
3.05–2.39	37.4		C-4 terminal units

**Table 3:** Identified compounds in TC-MeOH, TC-EtOAc, and TC-H_2_O extracts by HPLC HILIC-DAD-FLD-ESI-MS.

Tr	[M − H]^−^	Identification	Fragmentation	UV s (nm)	mg/g in TC-MeOH	mg/g in TC-EtOAc	mg/g in TC-H_2_O
14.6	447	Naringenin-7-*O*-glucuronide	MS ^2^ [447]: 271(100) MS ^3^ [271]: 151(100)-175(25) MS ^4^ [151]: 107(100)	200, 280	*	0.24 ± 0.06	0.006 ± 0.0003
16.0	463	Eriodictyol-7-*O*-glucuronide	MS ^2^ [463]: 287(100) MS ^3^ [287]: 151(100) MS ^4^ [151]: 107(100)	230, 280	0.57 ± 0.09	7.64 ± 0.8	0.48 ± 0.06
17.9	847	A-type proanthocyanidin trimer with one unit of (epi)afzelechin Isomer 1	MS^2^ [847]: 711(98)-559(100)-327(7)	280	0.95 ± 0.06	7.40 ± 0.4	0.06 ± 0.003
19.1	847	A-type proanthocyanidin trimer with one unit of (epi)afzelechin Isomer 2	MS ^2^ [847]: 711(92)-559(100) MS ^3^ [711]: 585(100)-559(75)-423(60) MS ^4^ [585]: 423(100) MS ^3^ [559]: 389(100) MS ^4^ [389]: 362(50)-345(100)-273(3)	280	8.96 ± 0,45	11.7 ± 2.1	0.05 ± 0.007
19.5	577	B-type procyanidin dimer	MS ^2^ [877]: 425(100)-407(60)-289(30) MS ^3^ [425]: 407(100)-273(10)-281(8) MS ^4^ [407]: 389(20)-339(30)-285(100)-281(98)-256(40)-269(20)-243(22)-213(10)	280	0.39 ± 0.07	11.13 ± 0.3	0.48 ± 0.02
20.3	461	Luteolin-7-*O*-glucuronide	MS ^2^ [461]: 285(100) MS ^3^ [285]: 257(45)-243(25)-241(90)-213(50)-199(100)-175(90)-151(35)	225, 280	2.13 ± 0.2	16.4 ± 1.2	1.25 ± 0.07
20.8	863	A-type procyanidin trimer	MS ^2^ [863]: 711(100)-573(50)-451(70)-411(70) MS ^3^ [711]: 559(100)-407(27) MS ^4^ [559]: 415(90)-327(60)-255(100)	280	9.73 ± 0, 91	38.69 ± 2.6	2.58 ± 0.21
22.4	1135	A-type proanthocyanidin tetramer with one unit of (epi)afzelechin	MS ^2^ [1135]: 999(70)-847(100)-707(70)-634(58)	280	25.2 ± 0.17	5.70 ± 1.8	0.09 ± 0.004
25.1	1151	A-type procyanidin tetramer	MS ^2^ [1151]: 1025(60)-863(100)-709(60)-573(25)	280	6.5 ± 0.76	0.44 ± 0.8	*
25.3	1424 [M − 2H]^2−^	B-type proanthocyanidin decamer with two units of (epi)afzelechin	MS ^2^ [1424]: 1271(100)	280	0.69 ± 0.09	4.74 ± 0.1	0.02 ± 0.005
26.1	1151 [M − 2H]^2−^	A-type procyanidin octamer	MS ^2^ [1151]: 863(42)-777(55)	280	8.97 ± 0.06	0.62 ± 0.05	0.09 ± 0.03
26.9	1438 [M−2H]^2−^	A-type procyanidin decamer with two A bonds	MS ^2^ [1438]: 1191(100)	280	11.4 ± 0.1	0.61 ± 0.04	0.013 ± 0.001
28.5	720 [M − 2H]^2−^	B-type procyanidin pentamer	MS ^2^ [720]: 643(100) MS ^3^ [643]: 559(65)-407(25)	280	5.67 ± 0.1	5.97 ± 0.08	0.09 ± 0.006
28.6	719 [M − 2H]^2−^	A-type procyanidin pentamer	MS ^2^ [719]: 567(50)-451(20)	280	10.8 ± 1.1	0.99 ± 0.09	0.07 ± 0.007
29.8	1440 [M − 2H]^2−^	B-type procyanidin decamer	MS ^2^ [1440]: 1313(100)-961(55)-817(70)	280	0.51 ± 0.2	2.79 ± 0.06	0.14 ± 0.004
33.0	864 [M − 2H]^2−^	B-type procyanidin esamer	MS ^2^ [864]: 779(90)-575(70)-532(75)-411(100)-289(20)	280	5.85 ± 0.4	5.44 ± 0.1	0.15 ± 0.003
34.3	1008 [M − 2H]^2−^	B-type procyanidin heptamer	MS ^2^ [1008]: 777(55)	280	9.87 ± 1.3	1.78 ± 0.03	0.08 ± 0.005

* detectable but not quantifiable.

**Table 4:** Quantitative results of total flavonoids, total procyanidin (PAC), and PACs divided on the basis of different degrees of polymerization in TC-MeOH, TC-EtOAc, and TC-H_2_O extracts.

Sample	Total Flavonoid (mg/g)	Total PAC (mg/g)	PAC Dimers (mg/g)	PAC Trimers (mg/g)	PAC Tetramers and Polymers (mg/g)
TC-MeOH	2.70 ± 0.05	105.49 ± 0.15	0.39 ± 0.01	19.64 ± 0.13	85.46 ± 0.16
TC-EtOAc	24.28 ± 0.15	98.00 ± 0.12	11.13 ± 0.15	57.79 ± 0.15	29.08 ± 0.15
TC-H_2_O	1.74 ± 0.05	3.91 ± 0.05	0.48 ± 0.01	2.69 ± 0.05	0.74 ± 0.02

**Table 5:** Results of total phenolic content and in vitro antioxidant assays on *T. coadunate* extracts.

Samples	Total Phenolic Content (mg GAE/g)	DPPH (mg TE/g)	ABTS (mg TE/g)	CUPRAC (mg TE/g)	FRAP (mg TE/g)	Metal chelating (mg EDTAE/g)	Phosphomolybdenum (mmol TE/g)
TC-EtOAc	276.70 ± 2.58 ^a^	948.59 ± 30.92 ^a^	1661.21 ± 9.01 ^a^	1510.63 ± 31.55 ^a^	931.18 ± 17.74 ^a^	na	6.32 ± 0.41 ^a^
TC-H_2_O	235.85 ± 1.82 ^b^	933.97 ± 12.12 ^a^	1269.30 ± 21.75 ^b^	1108.66 ± 4.44 ^b^	713.07 ± 11.98 ^b^	6.26 ± 0.73 ^a^	6.25 ± 0.18 ^a^
TC-MeOH	234.30 ± 0.99 ^b^	762.62 ± 34.65 ^b^	1097.10 ± 14.02 ^c^	1089.99 ± 6.42 ^b^	645.59 ± 4.83 ^c^	2.61 ± 0.34 ^b^	5.70 ± 0.59 ^a^

Values are reported as mean ± SD of three parallel experiments. ABTS: 2,2′-azino-bis(3-ethylbenzothiazoline-6-sulphonic acid); CUPRAC: cupric-reducing antioxidant; DPPH: 2,2-diphenyl-1-picrylhydrazyl; FRAP: ferric-reducing antioxidant power; GAE: gallic acid equivalent; TE: Trolox equivalent; EDTAE: EDTA equivalent; na: not active. Different superscripts indicate significant differences in the extracts (*p* < 0.05).

**Table 6:** Results of in vitro enzyme inhibition assays on *T. coadunata* extracts.

Samples	AChE Inhibition (mg GALAE/g)	BChE Inhibition (mg GALAE/g)	Tyrosinase Inhibition (mg KAE/g)	Amylase Inhibition (mmol ACAE/g)	Glucosidase Inhibition (mmol ACAE/g)
TC-EtOAc	6.22 ± 0.06 ^a^	9.82 ± 0.68 ^a^	153.89 ± 1.61 ^a^	1.50 ± 0.02 ^a^	5.46 ± 0.05 ^a^
TC-H_2_O	1.35 ± 0.03 ^c^	1.70 ± 0.67 ^c^	66.85 ± 1.22 ^c^	0.42 ± 0.04 ^c^	5.48 ± 0.01 ^a^
TC-MeOH	5.58 ± 0.10 ^b^	6.31 ± 0.71 ^b^	149.41 ± 0.96 ^b^	1.04 ± 0.05 ^b^	5.48 ± 0.01 ^a^

Values are reported as mean ± SD of three parallel experiments. GALAE: galantamine equivalent; ACAE: acarbose equivalent; KAE: kojic acid equivalent; AChE: acetylcholinesterase; BChE: butyrylcholinesterase. Different superscripts indicate significant differences in the extracts (*p* < 0.05).

**Table 7:** Cytotoxicity tests (concentration of compound inhibiting cell growth by 50% (IC_50_, µg/mL)) of TC-MeOH, TC-EtOAc, and TC-H_2_O extracts on 2008 and BxPC3 cell lines.

Samples	2008	BxPC3
TC-EtOAc	28, 7	12, 5
TC-H_2_O	>50	>50
TC-MeOH	>50	>50

## Supplementary Materials

The supplementary materials are available online.

## Abbreviations

ABTS	2:2′-azino-bis(3-ethylbenzothiazoline-6-sulphonic acid)
CUPRAC	cupric-reducing antioxidant
DPPH	2,2-diphenyl-1-picrylhydrazyl
FRAP	ferric-reducing antioxidant power
FLD	fluorescence detector
ESI-LC-DAD-MS	electrospray ionization source–liquid chromatography–diode array detector–mass spectrophotometry
NMR	nuclear magnetic resonance
PAC	procyanidin
TC-MeOH	*Tectaria coadunata* methanolic extract
TC-EtOAc	*T. coadunata* ethyl acetate extract
TC-H_2_O	*T. coadunata* water extract
GC-MS	gas chromatography–mass spectrophotometry
AChE	acetylcholinesterase
BuChE	butyrylcholinesterase
AD	Alzheimer’s disease
TLC	thin layer chromatography
TDDS	turbo detection data scanning
HSQC-DEPT	heteronuclear single quantum coherence spectroscopy–distortionless enhancement by polarization transfer
HMBC	heteronuclear multiple quantum coherence
COSY	correlation spectroscopy
