# Supplementary material for: Phytochemical Fingerprinting and In Vitro Bioassays of the Ethnomedicinal Fern Tectaria coadunata (J. Smith) C. Christensen from Central Nepal
Source: Molecules. 2019 Dec 5;24(24):4457. doi: 10.3390/molecules24244457 (PMC6943667; doi:10.3390/molecules24244457)
Supplement: Supplementary file 1 [file molecules-24-04457-s001.pdf]

Supplementary material

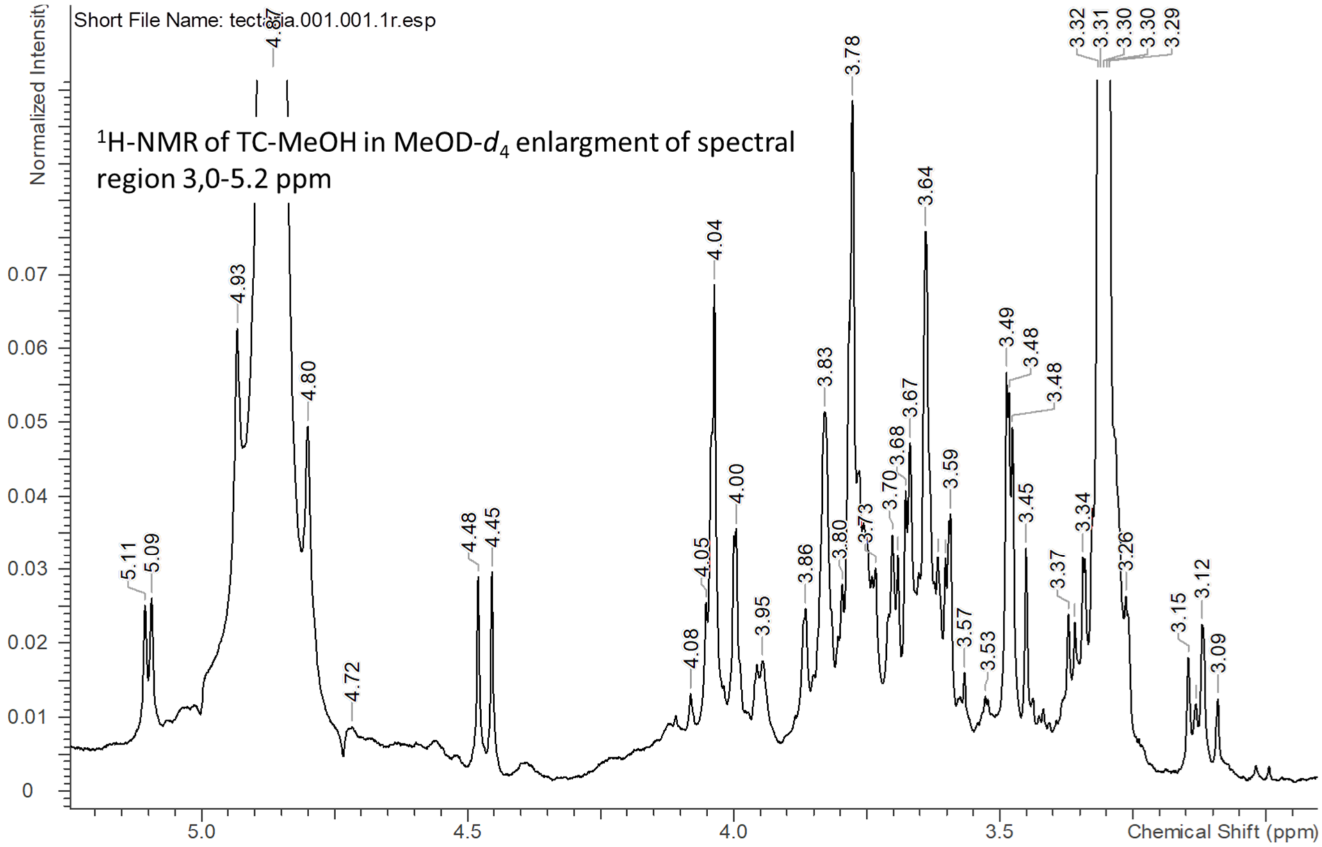

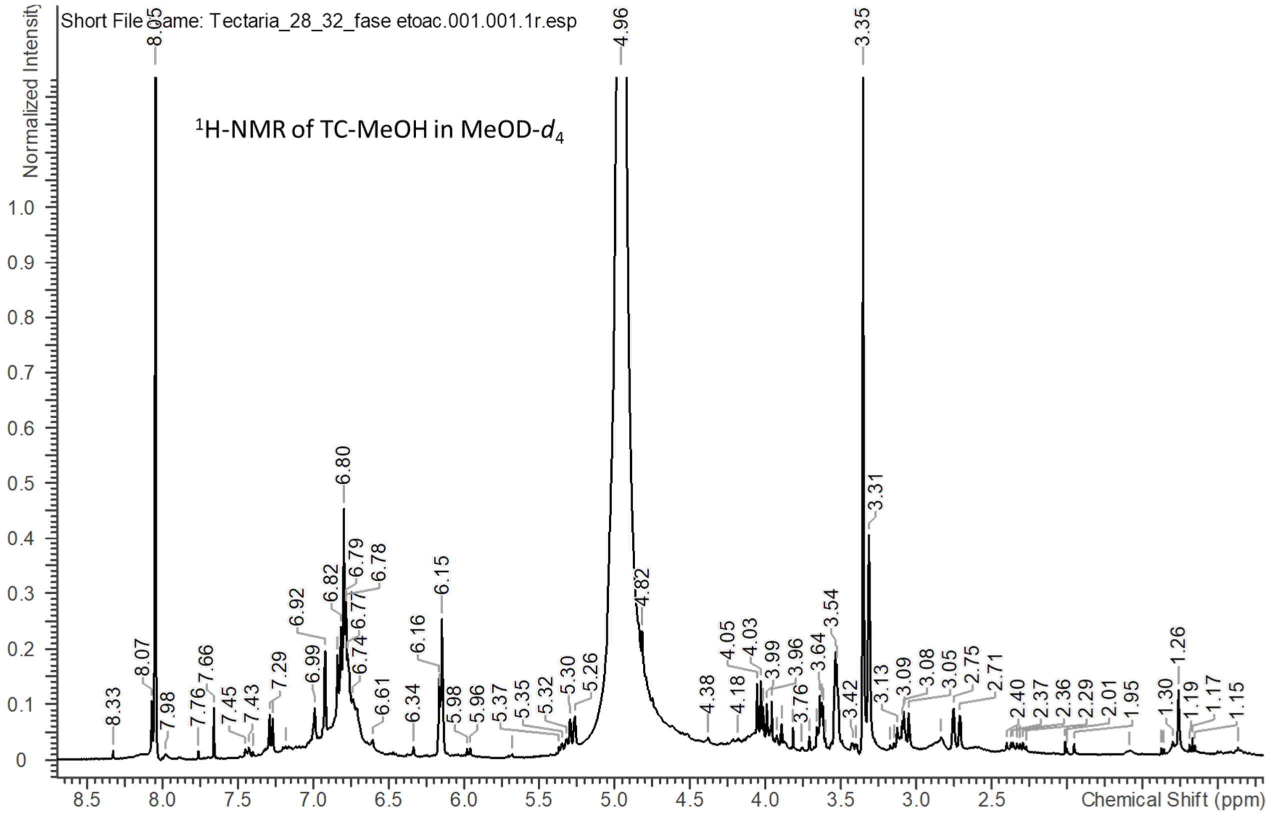

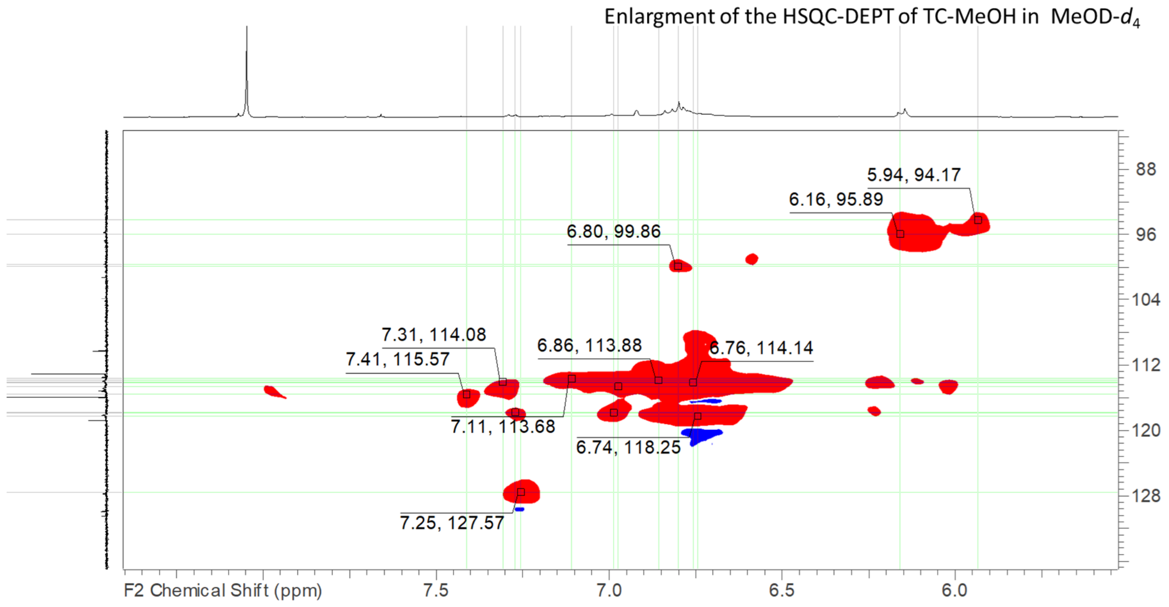
Enlargment of the HSQC-DEPT of TC-MeOH in MeOD-d_4
5.94, 94.17
6.16, 95.89
6.80, 99.86
7.31, 114.08
7.41, 115.57
6.86, 113.88
6.76, 114.14
7.11, 113.68
6.74, 118.25
7.25, 127.57
F2 Chemical Shift (ppm)

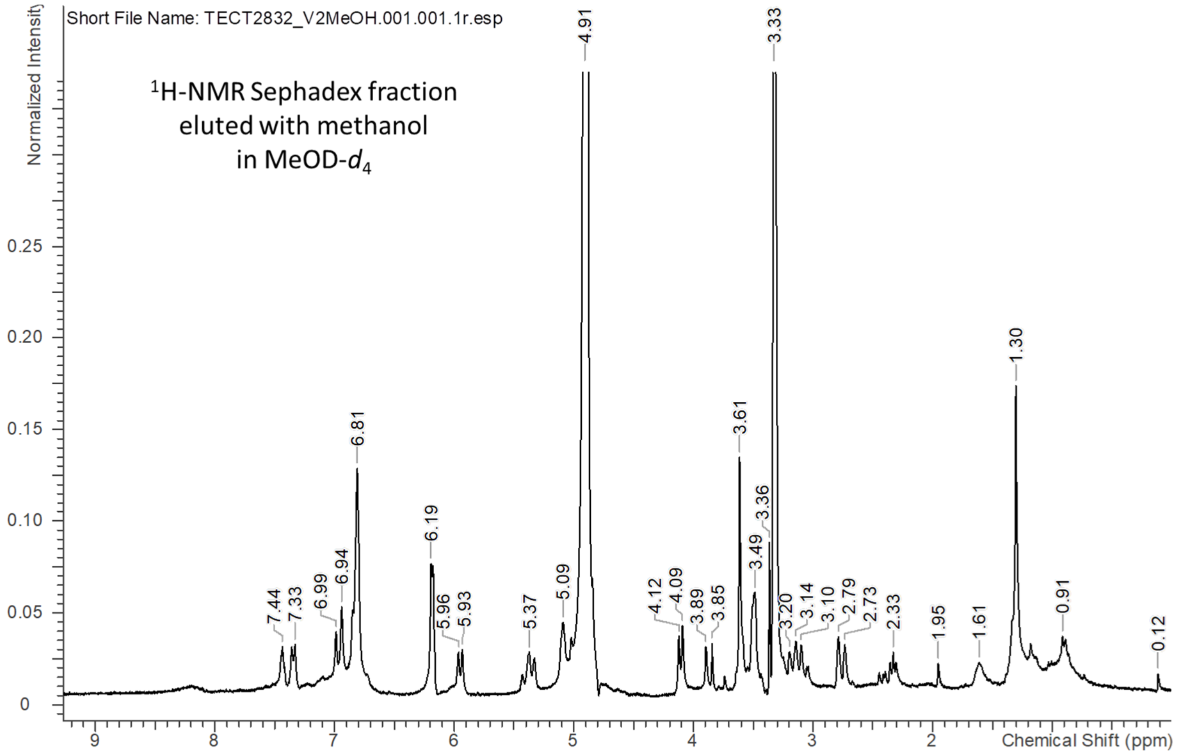
Short File Name: TECT2832_V2MeOH.001.001.1r.esp
^{1}H-NMR Sephadex fraction eluted with methanol in MeOD-d_4
Normalized Intensity
Chemical Shift (ppm)

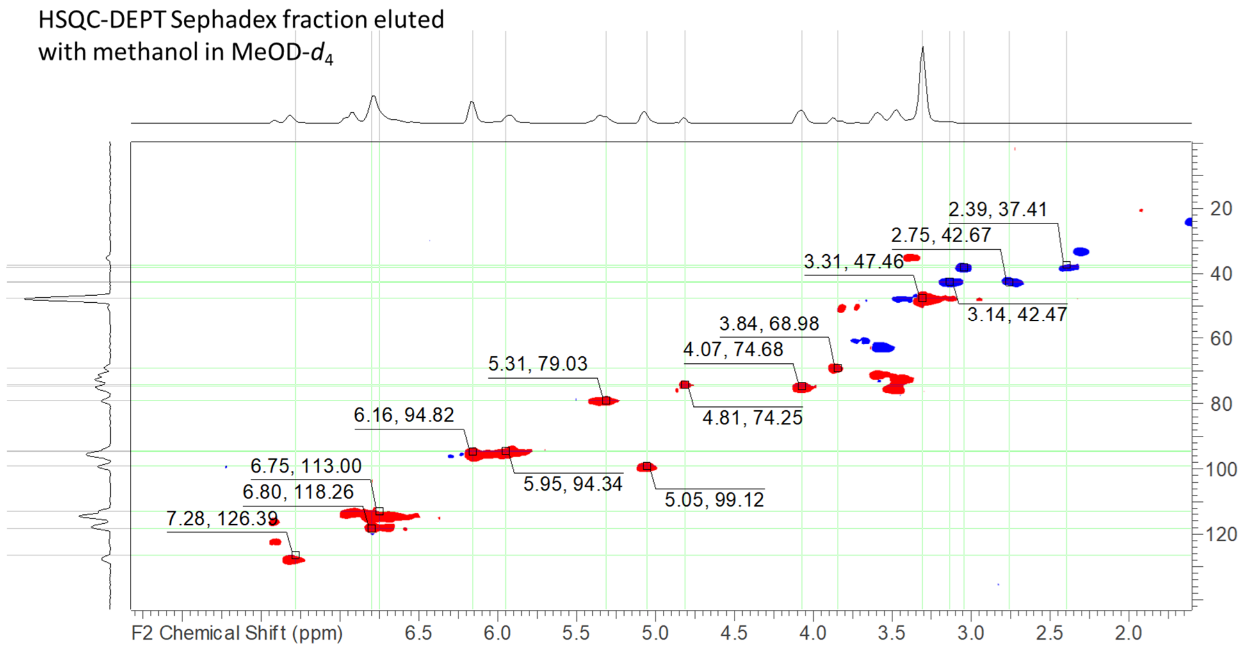

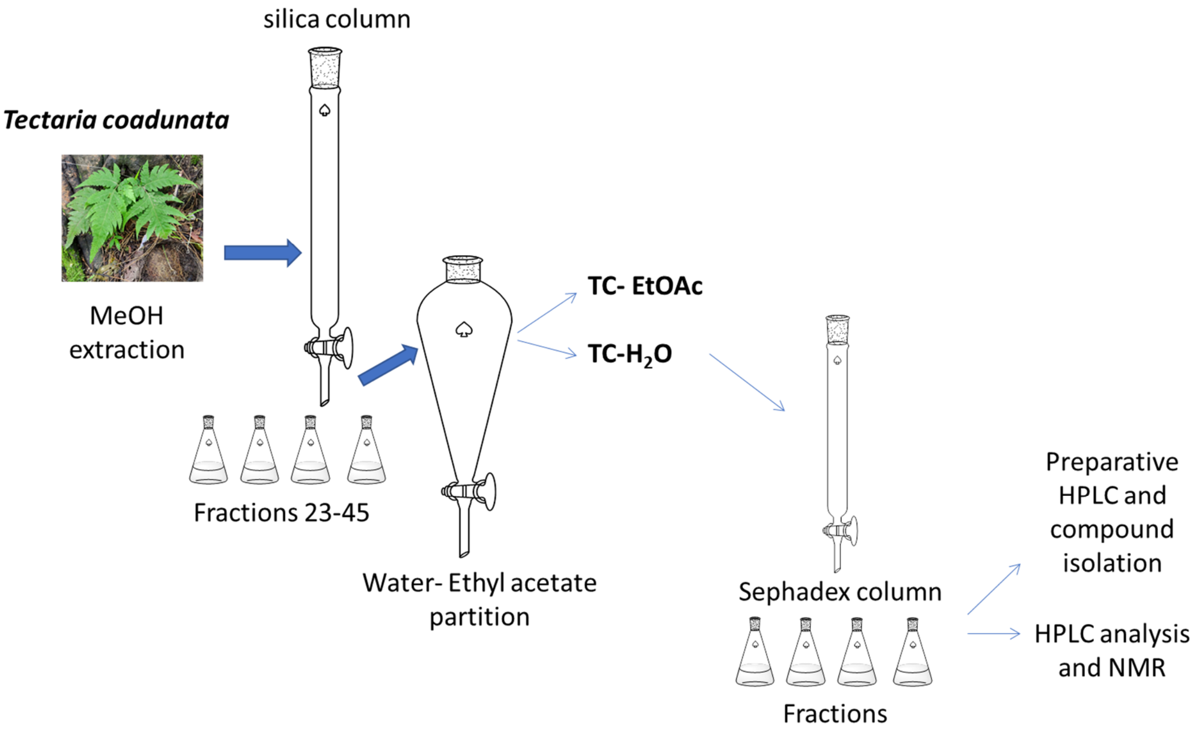

Schematic representation of extraction and fractionation procedures
